# A Cystine-Rich Whey Supplement (Immunocal^®^) Delays Disease Onset and Prevents Spinal Cord Glutathione Depletion in the hSOD1^G93A^ Mouse Model of Amyotrophic Lateral Sclerosis

**DOI:** 10.3390/antiox3040843

**Published:** 2014-12-12

**Authors:** Erika K. Ross, Aimee N. Winter, Heather M. Wilkins, Whitney A. Sumner, Nathan Duval, David Patterson, Daniel A. Linseman

**Affiliations:** 1 Department of Biological Sciences and Eleanor Roosevelt Institute, University of Denver, 2199 S. University Blvd., Denver, CO 80208, USA; E-Mails: Ross.Erika@mayo.edu (E.K.R.); Aimee.Winter@du.edu (A.N.W.); hwilkins@kumc.edu (H.M.W.); wsumner714@gmail.com (W.A.S.); nathan.duval@du.edu (N.D.); dpatter2@du.edu (D.P.); 2 Research Service, Veterans Affairs Medical Center, 1055 Clermont St., Denver, CO 80220, USA; 3 Division of Clinical Pharmacology and Toxicology, Department of Medicine and Neuroscience Program, University of Colorado Denver, 12700 E 19th Ave., Aurora, CO 80045, USA

**Keywords:** Immunocal^®^, whey protein, oxidative stress, glutathione, cysteine, amyotrophic lateral sclerosis

## Abstract

Depletion of the endogenous antioxidant, glutathione (GSH), underlies progression of the devastating neurodegenerative disease, amyotrophic lateral sclerosis (ALS). Thus, strategies aimed at elevating GSH may yield new therapeutics for ALS. Here, we investigated the effects of a unique non-denatured whey protein supplement, Immunocal^®^, in the transgenic Gly position 93 to Ala (G93A) mutant hSOD1 (hSOD1^G93A^) mouse model of ALS. Immunocal^®^ is rich in the GSH precursor, cystine, and is therefore capable of bolstering GSH content. Transgenic hSOD1^G93A^ mice receiving Immunocal^®^ displayed a significant delay in disease onset compared to untreated hSOD1^G93A^ controls. Additionally, Immunocal^®^ treatment significantly decreased the rate of decline in grip strength and prevented disease-associated reductions in whole blood and spinal cord tissue GSH levels in end-stage hSOD1^G93A^ mice. However, Immunocal^®^ did not extend survival, likely due to its inability to preserve the mitochondrial GSH pool in spinal cord. Combination treatment with Immunocal^®^ and the anti-glutamatergic compound, riluzole, delayed disease onset and extended survival in hSOD1^G93A^ mice. These findings demonstrate that sustaining tissue GSH with Immunocal^®^ only modestly delays disease onset and slows the loss of skeletal muscle strength in hSOD1^G93A^ mice. Moreover, the inability of Immunocal^®^ to rescue mitochondrial GSH in spinal cord provides a possible mechanism for its lack of effect on survival and is a limiting factor in the potential utility of this supplement as a therapeutic for ALS.

## 1. Introduction

Amyotrophic lateral sclerosis (ALS) is the most common adult-onset motor neuron disease. It is characterized clinically by progressive skeletal muscle weakness, atrophy and paralysis, ultimately leading to respiratory failure and death within two to five years of diagnosis. ALS is characterized pathologically by a progressive loss of motor neurons in the cortex, brainstem and spinal cord [1]. Though its underlying cause remains elusive, oxidative damage due to aberrant production of reactive oxygen species (ROS) and associated mitochondrial dysfunction play key roles in motor neuron death [2,3,4]. Only approximately 10% of ALS cases are considered familial, with the remaining 90% characterized as sporadic with no known cause. Mutations in Cu/Zn-superoxide dismutase (SOD1) are one of the most common causes of familial ALS. Nearly 150 mutant forms of SOD1 have been identified in ALS patients, and these are collectively responsible for approximately 20% of all cases of familial disease [5].

Mutant forms of SOD1 have been implicated in the aberrant generation of ROS at the mitochondria and subsequent mitochondrial dysfunction, which underlie ALS disease pathology. The mutant form of SOD1 harboring a glycine to alanine mutation at position 93 (SOD1^G93A^) in particular, displays a gain of toxic function that is characterized by an increased generation of mitochondrial ROS [6]. Mutant SOD1 also accumulates at mitochondria and triggers a shift in the redox state of these organelles [7]. Moreover, crossing of transgenic hSOD1^G93A^ mutant mice with mice heterozygous for the deletion of the mitochondrial-specific SOD2 gene significantly exacerbates motor neuron pathology and reduces lifespan in this model of familial ALS, further indicating a central role for mitochondrial oxidative stress (MOS) in disease pathogenesis [8].

Glutathione (GSH) is an endogenous tri-peptide antioxidant that plays a critical role in detoxifying ROS generated within cells, particularly at mitochondria [9]. Mitochondria possess a discrete pool of GSH, which is critical for the prevention of intrinsic apoptosis and other cell death pathways [10,11]. Decreased levels of GSH have been observed in erythrocytes of patients with sporadic ALS and are correlated with disease progression, indicating that depletion of this key antioxidant is likely one underlying factor in disease progression [12]. Moreover, GSH depletion has been observed in vivo in whole spinal cord of end-stage hSOD1^G93A^ mice, and more specifically, the ratio of reduced GSH-to-oxidized glutathione disulfide (GSSG) in spinal cord mitochondria of mutant SOD1 mice is significantly diminished, indicative of MOS and concurrent GSH depletion [13,14].

Because GSH depletion has been implicated in the pathogenesis of both sporadic and familial forms of ALS, treatments aimed at preserving GSH levels may lead to new potential therapeutic options for ALS patients. Treatment with agents, such as *N*-acetylcysteine (NAC) and GSH-monoethyl ester (GSH-MEE), has shown some promise in pre-clinical ALS mouse models; however, the relatively low bioavailability of these agents, and in the case of GSH-MEE, the necessity for central delivery of the drug, limit the utility of these agents for treatment of human patients [15,16]. Similarly, NAC, though it can be orally administered, has very low bioavailability and often requires intravenous administration to increase circulating levels of this compound [15]. Immunocal^®^ (Immunotec Inc., Vaudreuil-Dorion, Quebec, Canada) is a whey protein supplement that contains abundant amounts of cystine, a cysteine precursor, due to its unique non-denaturing preparation. As cysteine is required for the rate limiting step of GSH synthesis, supplementation with cystine-rich compounds is an effective way to enhance de novo tissue GSH synthesis. Cysteine itself is rapidly catabolized in the GI tract and is capable of producing toxicity when administered in a pure form [17]. On the other hand, cystine acts as a cysteine delivery system, which is rapidly reduced into two cysteine molecules upon uptake by target cells, significantly limiting toxicity. Furthermore, Immunocal^®^ displays significantly higher bioavailability than NAC when administered orally [18,19].

Immunocal^®^ was initially developed as a nutritional supplement to increase immune system function, and it is one of only a handful of nutritional supplements that are included in the Physician’s Desk Reference (see Table 1 for composition) [20]. This supplement was found to have beneficial effects in clinical disorders for which oxidative stress is a significant underlying factor, including HIV infection and cystic fibrosis [21,22]. Based on these studies, Immunocal^®^ holds significant potential as an agent to bolster GSH levels and, thus, may provide a novel therapeutic approach for the treatment of neurodegenerative diseases for which the underlying pathology involves significant oxidative stress. Here, we sought to determine its effects in the hSOD1^G93A^ mutant mouse model of familial ALS.

**Table 1:** Immunocal^®^ constituents by mass per one packet of supplement ^a,b^.

Component	Supplement Content	% of Total Supplement
Whey proteins (serum albumin, α lactalbumin and lactoferrin)	8.8–9.2 g	88%–92%
Fat	~0.05 g	<0.5%
Lactose	~0.15 g	<1.5%
Minerals (Ca, Na)	~0.30 g	<3.0%
Moisture	0.5 g	~5%

^a^ One packet of Immunocal^®^ contains approximately 10 g of protein supplement (one serving) in fine powder form and 40 calories per serving. ^b^ Constituents appear as determined by [22].

## 2. Experimental Section

### 2.1. Mouse Model of ALS

All animal procedures were performed according to a protocol approved by the University of Denver Institutional Animal Care and Use Committee. FVB-Tg (SOD1-G93A) mice with the toxic gain of function SOD1 Gly position 93 to Ala mutation (G93A) were obtained from The Jackson Laboratory (Bar Harbor, ME, USA). The colony was maintained and bred in the animal facility at the University of Denver. Animal genotyping was done by the third party company, Transnetyx (Cordova, TN, USA). Littermates of both sexes (approximately equal numbers of males and females) were included in each group, with an age- and sex-matched non-transgenic (NonTG) mouse as the control, with the exception of the treatment groups containing mice receiving Immunocal^®^ supplement alone. These groups were predominately composed of male hSOD^G93A^ mice, which typically show a slightly shorter life span than their female counterparts (Table 2 and Table 3). No significant gender differences in the age of disease onset were observed in our colony (Table 2). For experiments assessing the effects of Immunocal^®^ alone, animals receiving supplementation had ad libitum access to a 3.3% solution of Immunocal^®^ in drinking water beginning at 60 days of age. For experiments utilizing Immunocal^®^ in conjunction with the anti-glutamatergic drug, riluzole, animals receiving Immunocal^®^ supplementation were dosed twice daily, five days a week, with 0.25 mL of a solution containing 3.3% Immunocal^®^ in sterile drinking water via oral gavage, which corresponds to a dose of approximately 660 mg/kg/day (based on a 25-g mouse). The typical daily dose of Immunocal^®^ when taken as a nutritional supplement is two 10-g packets per day or approximately 285 mg/kg/day (based on a 70-kg person). Riluzole was administered ad libitum in drinking water at a dose of 100 μg/mL of water.

**Table 2:** Gender effects on disease onset and survival in amyotrophic lateral sclerosis (ALS) mice.

G93A SOD1 Transgenic	Males (*n* = 11)	Females (*n* = 12)
Onset (days ± SEM)	90 ± 1.0	92.8 ± 1.4
Survival (days ± SEM)	123.3 ± 2.2	128.6 ± 3.1

G93A, Gly position 93 to Ala; SOD1, Cu/Zn-superoxide dismutase.

**Table 3:** Gender distribution of treatment groups and statistical analysis of disease onset.

Group Number(s)	Treatment	Females	Males	Total *N*	Mean Onset (days)	SEM
1	NonTG	11	13	24		
2	G93A SOD1 Control	12	11	23	91	1
3	ICAL (ad libitum)	5	8	13	99 **	1
4	ICAL (oral gavage)	1	4	5	98 *	3
5	ICAL (oral gavage) + riluzole	5	5	10	101 ***	2

NonTG, Non-transgenic; ICAL, Immunocal^®^. * *p* < 0.05, ** *p* < 0.01, *** *p* < 0.001 *vs.* G93A SOD1 control (Student’s *t*-test).

The decision to start Immunocal^®^ supplementation at 60 days of age was based upon accepted practices for designing studies using ALS mouse models as recommended by the Jackson Laboratory [23]. Briefly, when working with mouse models of ALS, preclinical studies are conducted by first treating mice with a potential therapeutic agent between 50 and 70 days of age. If this pre-symptomatic treatment yields a robust therapeutic effect, a second study can be conducted to analyze the efficacy of administering the agent at or after disease onset. Seeing that Immunocal^®^ administration produced a very modest positive effect when given pre-symptomatically, we did assess its applicability at disease onset (approximately 90 days of age) in a small number of mice. However, Immunocal^®^ administration beginning at this stage of the disease did not appear to affect either disease progression or survival (data not shown).

### 2.2. Clinical Tests

Paw grip endurance (PaGE) testing was performed, and scores were determined by the latency of the animal’s hind limbs to detach from a conventional housing wire cage lid after inversion. Briefly, mice were placed on top of the wire cage lid and given a few seconds to acclimate before the cage lid was gently inverted in one smooth motion, prompting the mouse to grip the wire. Care was taken to ensure that no jostling occurred during inversion, which might have caused the mouse to fall. The lid was held still and suspended while the mouse gripped the wire lid, and a stop watch was used to determine the point at which the mouse’s hind limbs detached from the wire lid. Time was stopped the moment the mouse’s hind limbs detached from the cage lid or after 30 s with no detachment, and the score was recorded as the latency to fall. Mice were given three scored attempts. Final scores were recorded as the average of these three trials for each time point, with a maximum possible score of 30 s. The PaGE test was carried out twice weekly [24]. Scores are reported as the average of all animals’ final scores ± SEM for each time point. Time points correspond to the age of the animal at the time of testing and are represented as a span of 5 days due to the fact that multiple groups of animals having slightly different ages were tested concomitantly during the twice-weekly testing regimen. Animals were weighed every other day starting on Day 30. Disease onset was determined by the appearance of hind limb trembling, inability to splay the hind legs or failure of the mouse to extend either hind leg when suspended by its tail, which would indicate the first appearance of muscle weakness or paralysis. Though onset was not determined in a blinded fashion, at least two investigators confirmed the appearance of neurological symptoms of the disease in an effort to lessen subjectivity when determining disease onset. We assessed disease onset in the manner described above daily beginning at 60 days of age. Determination of disease onset by monitoring mice harboring ALS causing mutations for neurological deficits, such as the inability to splay hind limbs when suspended by the tail, is a well-accepted and common way to determine disease onset [23,25]. Animals were euthanized at the end-stage of disease, which is defined as the point at which the mouse is no longer capable of righting itself or performing regular motor tasks that would be required to reach food and water. This occurs due to extensive paralysis of the hind limbs and weakness in the upper extremities as a result of disease progression. The end-stage of the disease was determined by a failure of the animal to right itself to sternum within 20 s upon being placed on its side.

### 2.3. Tissue Processing

Tissues, including cerebral cortex, lumbar spinal cord and trunk blood, were harvested from end-stage hSOD1^G93A^ or age and sex-matched NonTG mice post-isoflurane overdose and were immediately frozen in liquid nitrogen (−196 °C). For HPLC-EC analysis, frozen trunk blood was weighed and 300 μL of HCL was added to the sample and vortexed rapidly. It was next centrifuged at 10,000× *g* for 20 min at 4 °C. The supernatant was removed and added to a new tube containing 10% perchloric acid (PCA), which was centrifuged for 10 min at 10,000× *g* at 4 °C. A clear supernatant was removed and filtered. Frozen lumbar spinal cord was weighed and added to 1 mL of 10% PCA before vortexing 3 times for 15 s each time. The supernatant was removed and filtered. Spinal cord GSH values were normalized to protein concentrations, which were determined by a commercially-available protein assay kit (BCA, Thermo Scientific, Rockford, IL, USA). Samples were analyzed in triplicate, and an additional sample was spiked with reduced GSH and glutathione disulfide (GSSG) to verify peak identification.

### 2.4. High Performance Liquid Chromatography with Electrochemical Detection

GSH and GSSG in samples and known standards were separated by reverse phase HPLC on a C18 bonded silica column at 35 °C (3 μm, 3 × 150 mm for whole blood, 3 μm, 3 × 250 mm for lumbar spinal cord) from Dionex Inc. (Sunnyvale, CA, USA). Analytes were detected using a CoulArray^®^ detector (model 5600A, ESA; ESA Inc., Chelmsford, MA, USA) on three coulometric detector modules. Each electrochemical cell contains four coulometric detectors in series set to a range of potentials from 0 to 900 mV at increments of 100 mV. The mobile phase consisted of 125 mmol/L sodium phosphate and 1% HPLC grade methanol (Fisher Scientific, Pittsburg, PA, USA) in water, pH 2.8 [26]. The flow rate was set to 0.4 mL/min for all samples. CoulArray software was used for baseline correction and analysis.

### 2.5. Isolation of Mitochondria from Lumbar Spinal Cord

Mitochondria were isolated as previously described by Liang and Patel [27]. Briefly, lumbar spinal cord for each individual animal was dounce homogenized in 2 mL of mitochondrial isolation buffer (0.064 M sucrose, 2 mM EDTA, 20 mM Tris-HCl, pH 7.4). Samples were placed in an equal volume of 24% Percoll gradient and centrifuged at 4 °C for 5 min, 16,000 rpm to make a 12% Percoll gradient. Two milliliters of supernatant were then added to a Percoll gradient containing a bottom layer of 2 mL 40% Percoll and a top layer of 2 mL 19% Percoll. Samples were spun at 4 °C for 10 min, 16,000 rpm. The mitochondrial layer was removed (2 mL of the layer between the 19% and 40% gradients, respectively), washed with mitochondrial buffer at a dilution of 4:1 and spun at 4 °C for 10 min at 11,820 rpm. The supernatant was discarded, and the pellets were washed a second time with 4.5 mL of mitochondrial isolation buffer and 0.5 mL of mitochondrial isolation buffer containing 5 mg/mL BSA, then spun at 4 °C for 10 min at 7500 rpm. Mitochondrial pellets were then re-suspended in 100 μL of a mitochondrial buffer containing 130 mM KCl, 4 mM Tris-HCl, 5 mM sodium pyruvate, 5 mM sodium succinate and 1 mM EGTA, pH 7.4.

### 2.6. Mitochondrial GSH Loading and Measurement of GSH Levels in Isolated Mitochondria

For loading experiments, isolated mitochondria from either NonTG or hSOD1^G93A^ mice were incubated with 2 mM GSH at 37 °C, 300 rpm for 4 h after which mitochondria were washed three times, and total GSH was measured. A sample of unloaded mitochondria was frozen immediately after isolation to serve as an unloaded control. Values for this sample were subtracted from total GSH following loading to determine the amount of GSH loaded per hour. Total mitochondrial glutathione (both GSH and GSSG) for all experiments using isolated mitochondria was measured using a colorimetric kinetic assay kit from Oxford Biomedical (Rochester Hills, MI, USA) and was performed per the manufacturer’s instructions. All GSH measurements were normalized to protein concentration.

### 2.7. Statistical Analysis

Measurements of GSH were performed in duplicate and repeated a minimum of three times. Data are reported as the mean ± SEM. Statistical significance was analyzed with one-way analysis of variance (ANOVA) followed by a post hoc Tukey’s test, or using a paired Student’s *t*-test when two matched sets of data were compared. PaGE measurements of grip strength data were compared between groups at each time point using one-way ANOVA and a post hoc Tukey’s test. Survival and onset data were analyzed with Kaplan-Meier curves and a log rank test.

## 3. Results

### 3.1. Immunocal^®^ Delays Disease Onset, but Does Not Extend Lifespan in the hSOD1^G93A^ Mouse Model of ALS

**Figure 1:**
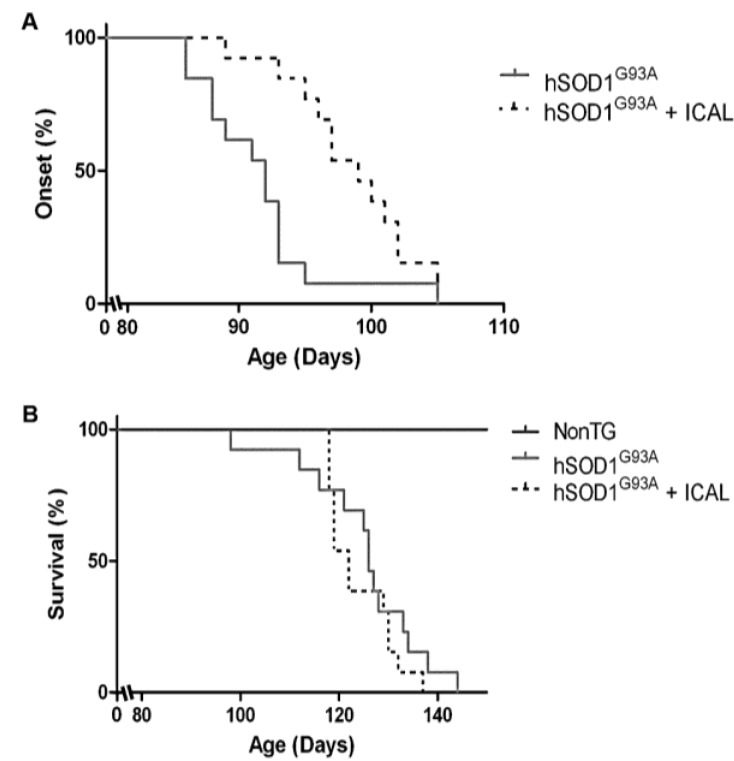
Immunocal^®^ delays clinical onset in hSOD1^G93A^ mice. (**A**) hSOD1^G93A^ mice receiving Immunocal^®^ ad libitum beginning at 60 days of age (pre-symptomatically) displayed a delay in disease onset and clinical decline compared to untreated mutant mice (*n* = 13). Onset curves are significantly different (*p* < 0.001) as determined by the Gehan–Breslow–Wilcoxon test. (**B**) Median survival is not significantly different between hSOD1^G93A^ mice receiving Immunocal^®^ ad libitum and untreated mutant mice (*n* = 13).

Here, we investigated the effects of Immunocal^®^ supplementation in the hSOD1^G93A^ mouse model of ALS. Beginning at 60 days-of-age, mice received Immunocal^®^ ad libitum in drinking water. Those mice receiving Immunocal^®^ displayed a significant delay in clinical onset of disease of approximately seven days (7.0 ± 1.1 days) with onset occurring on average at 98.5 ± 1.1 days of age in comparison to untreated hSOD1^G93A^ mice, which had an average disease onset of 91.6 ± 0.9 days of age (Figure 1A). However, treatment with Immunocal^®^ did not produce any measurable difference in lifespan. Mice receiving Immunocal^®^ displayed an average age of 125.2 ± 2.4 days at the end-stage in comparison to untreated hSOD1^G93A^ mice, which reached the end-stage at 124.1 ± 1.5 days of age (Figure 1B).

**Figure 2:**
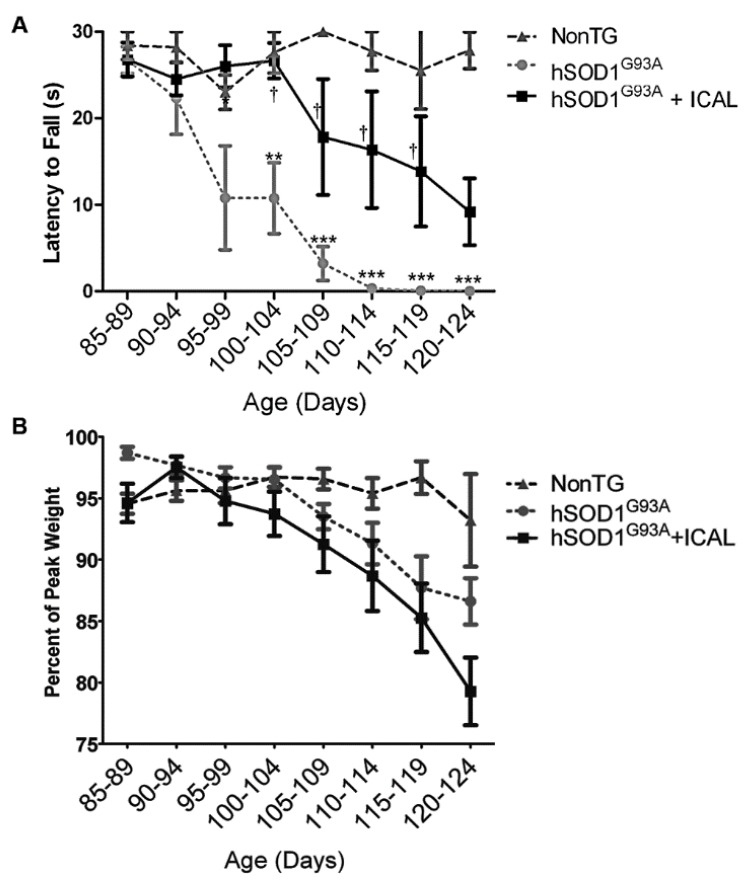
Immunocal^®^ diminishes the rate of decline in grip strength in hSOD1^G93A^ mice. (**A**) The paw grip endurance (PaGE) hanging wire test expressed as latency to fall at indicated ages (*n* = 13); (**B**) body weight of wild-type and hSOD1^G93A^ mice expressed as the percent of peak body weight. All data are represented as the mean ± SEM. *** indicates *p* < 0.001 compared to non-transgenic (NonTG) mice; ** indicates *p* < 0.01 compared to NonTG; **^†^** indicates *p* < 0.05 compared to untreated hSOD1^G93A^ mice (one-way ANOVA with a post hoc Tukey’s test conducted for each time point).

Mice were visually assessed for deficits in motor function and coordination daily and by the PaGE hanging wire grip test twice weekly. PaGE testing revealed a statistically significant decrease in the rate of decline in grip strength between 100 days and 120 days of age when Immunocal^®^-treated mice were compared to hSOD1^G93A^ mice that did not receive supplementation (Figure 2A). Despite retaining muscle strength, Immunocal^®^ did not show any ability to preserve body weight in transgenic mice receiving the supplement (Figure 2B). Thus, oral treatment with Immunocal^®^ preserved grip strength and modestly delayed the onset of motor dysfunction in this mouse model of familial ALS.

### 3.2. Immunocal^®^ Prevents GSH Depletion in Whole Blood and Lumbar Spinal Cord Tissue of hSOD1^G93A^ Mice

**Figure 3:**
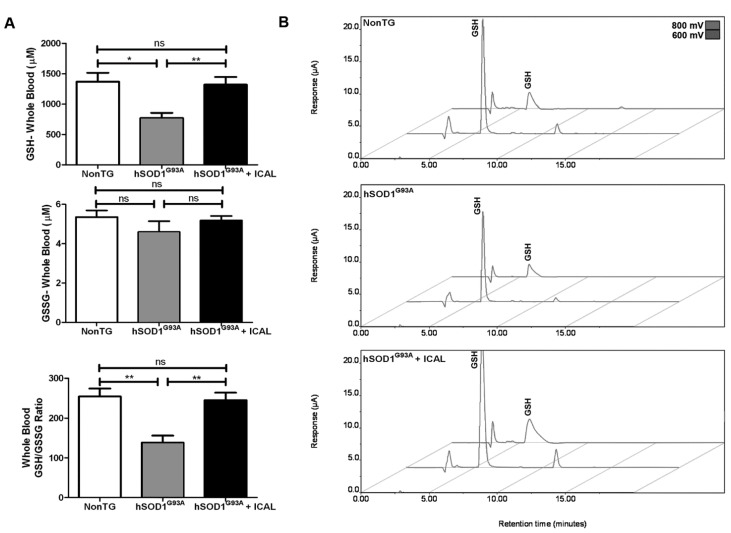
HPLC-EC detection reveals whole blood GSH is preserved in hSOD1^G93A^ mice receiving Immunocal^®^. (**A**) Mean GSH and glutathione disulfide (GSSG) concentrations and GSH/GSSG ratios from the whole blood of end-stage trial animals determined using HPLC-EC detection, *n* = 13. ** indicates *p* < 0.01; * indicates *p* < 0.05; ns indicates no significant difference. (**B**) Representative HPLC-EC chromatograms from the whole blood of end-stage animals; labeled peaks indicate GSH oxidizing at 600 and 800 mV.

The neuroprotective effects of Immunocal^®^ observed in vitro are largely due to its capacity to enhance GSH synthesis by providing the key precursors, cystine and cysteine. To investigate this potential mechanism of action *in vivo*, whole blood and lumbar spinal cord tissue were collected from end-stage hSOD1^G93A^ mice and analyzed for reduced GSH and oxidized GSSG using HPLC-EC. We found that hSOD1^G93A^ mice that did not receive supplementation displayed a significant decrease in whole blood GSH when compared to NonTG littermate controls. Levels of GSSG in whole blood did not significantly change; however, the ratio of GSH-to-GSSG in whole blood was significantly decreased in hSOD1^G93A^ mice compared to NonTG controls. Both the decreases in GSH and the reduction in the GSH/GSSG ratio were prevented in hSOD1^G93A^ mice receiving Immunocal^®^ (Figure 3A). Representative HPLC chromatograms from these animals are shown in Figure 3B.

**Figure 4:**
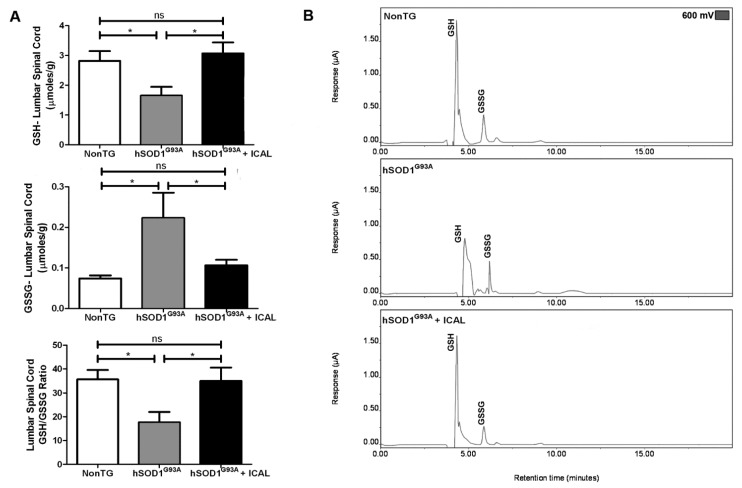
HPLC-EC detection reveals lumbar spinal cord GSH is maintained in hSOD1^G93A^ mice receiving Immunocal^®^. (**A**) Mean GSH and GSSG concentrations and GSH/GSSG ratios from the lumbar spinal cord of end-stage trial animals determined using HPLC-EC detection, *n* = 17. * indicates *p* < 0.05; ns indicates no significant difference. (**B**) Representative HPLC-EC chromatograms from the lumbar spinal cord of end-stage animals; labeled peaks indicate GSH and GSSG oxidizing at 600 mV.

Interestingly, we found that a similar pattern also existed for GSH levels in the lumbar spinal cord tissue of study animals. Spinal cord tissue GSH levels were decreased by approximately 50%; GSSG levels were elevated two-fold; and the ratio of GSH-to-GSSG was significantly decreased in hSOD1^G93A^ mice compared to NonTG controls. Immunocal^®^ treatment prevented these changes in GSH and GSSG and maintained the GSH/GSSG ratio at a level that was indistinguishable from NonTG controls (Figure 4A). Representative chromatograms from these animals are displayed in Figure 4B. These results indicate that the preservation of whole blood and spinal cord tissue GSH levels positively correlates with the beneficial effects of Immunocal^®^ supplementation in the mutant hSOD1^G93A^ mouse model of ALS.

### 3.3. Immunocal^®^ Does Not Prevent Depletion of Mitochondrial GSH in Lumbar Spinal Cord of hSOD1^G93A^ Mice

Although the ability of Immunocal^®^ to preserve total GSH levels in whole blood and lumbar spinal cord tissue of hSOD1^G93A^ mutant mice correlates with preserved grip strength and delayed disease onset, the underlying reason for the lack of effect on survival with Immunocal^®^ supplementation remained unclear. We have previously shown that specific deficits in mitochondrial GSH levels significantly sensitize neurons to oxidative insult [28]. Furthermore, recent evidence demonstrates that the GSH-to-GSSG ratio is significantly reduced in spinal cord mitochondria of mutant SOD1 mice, indicative of MOS and mitochondrial GSH depletion [14]. As MOS is a key feature of ALS disease pathology, we hypothesized that although Immunocal^®^ was able to bolster spinal cord tissue GSH, it may not have sustained mitochondrial GSH levels and, thus, would be unable to prevent MOS.

**Figure 5:**
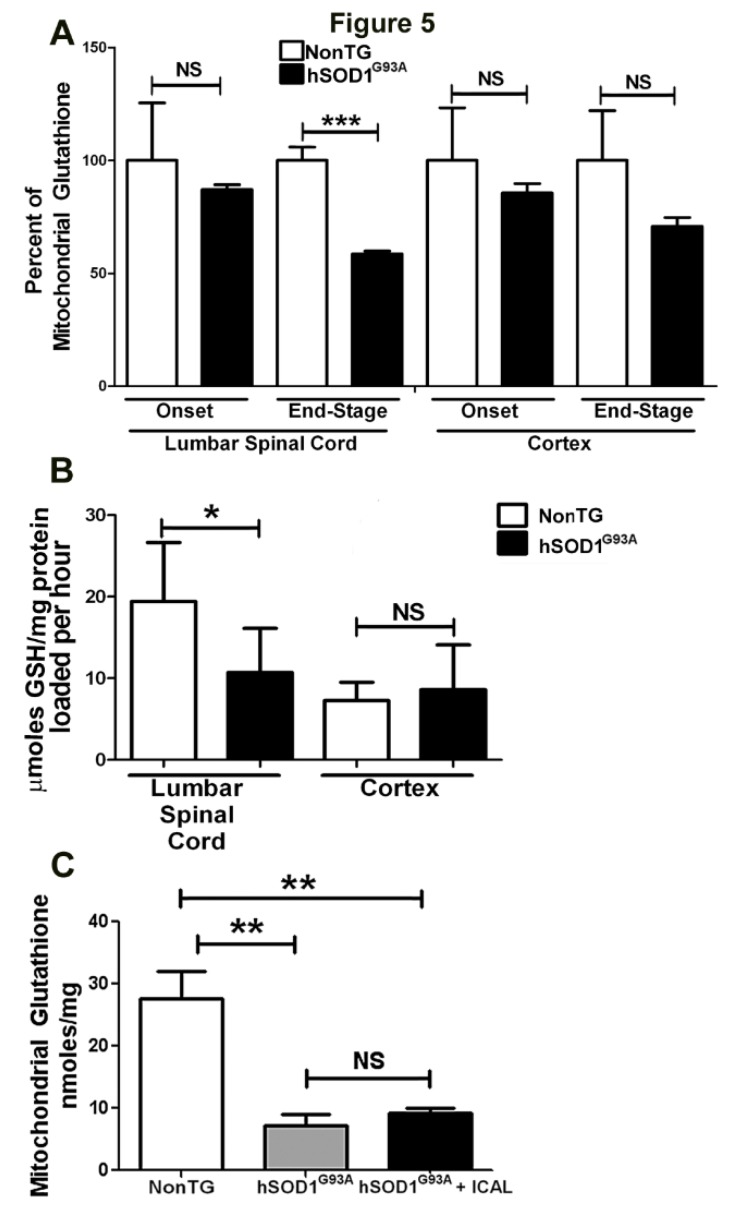
Spinal cord mitochondrial GSH levels and mitochondrial GSH transport are significantly diminished in hSOD1^G93A^ mice. (**A**) Mitochondria were isolated from NonTG and hSOD1^G93A^ mice at onset (~90 days old) and end-stage (~120 days old) from lumbar spinal cord and whole cortex, and total GSH was measured. All measurements were normalized to protein and represented as a percent of NonTG. *** indicates *p* < 0.001; NS indicates no significant difference, as determined by an unpaired Student’s *t*-test, *n* = 4. Error bars indicate SEM. (**B**) Mitochondria were isolated from lumbar spinal cord and cortex of end-stage (~120 days old) hSOD1^G93A^ and age-matched NonTG mice, as described in (**A**). Isolated mitochondria were incubated with 2 mM GSH at 37 °C, 300 rpm for 4 h, after which mitochondria were washed 3× and total GSH was measured. All measurements were normalized to protein and represented as μmoles GSH/mg of mitochondrial protein loaded per hour. * indicates *p* < 0.05; NS indicates no significant difference, as determined by a paired Student’s *t*-test, *n* = 7. Error bars indicated SEM. (**C**) Mitochondria were isolated from lumbar spinal cord as described in (**A**) from untreated end-stage (~120 days old) hSOD1^G93A^ mice, hSOD1^G93A^ mice treated with Immunocal^®^ (hSOD1^G93A^ + ICAL) and age-matched NonTG mice. Total GSH was measured. All measurements of total GSH are normalized to protein and represented as nmols/mg of protein. ** indicates *p* < 0.01; NS indicates no significant difference, as determined using a one-way ANOVA with a post hoc Tukey’s test, *n* = 4. Error bars indicate SEM.

To test this hypothesis, mitochondria were isolated from the cortex and lumbar spinal cord of hSOD1^G93A^ mice at the end-stage of disease, and total mitochondrial GSH levels were assessed. In comparison to their NonTG counterparts, hSOD1^G93A^ mice displayed a significant decrease in total mitochondrial GSH in lumbar spinal cord at the end-stage of disease (Figure 5A), consistent with prior reports of a reduced GSH to GSSG ratio; however, no deficit was observed at disease onset [14]. A similar trend was observed for mitochondria isolated from the cortex of mutant animals, but did not reach statistical significance. This observation encouraged further investigation into the mechanism by which mitochondrial GSH is depleted in hSOD1^G93A^ mice. Because mitochondria lack the enzymes necessary to synthesize GSH on their own, de novo synthesis takes place in the cytosol, and GSH is subsequently transported into the mitochondria [10]. Thus, we analyzed the ability of mitochondria isolated from the spinal cord and cortex of end-stage mutant mice and their NonTG littermates to take up GSH in vitro over time. Intriguingly, mitochondria isolated from hSOD1^G93A^ mutant spinal cord showed a marked decrease in GSH loading efficiency in comparison to NonTG controls (Figure 5B). This effect was not observed in mitochondria isolated from cortex (Figure 5B). We next examined the total GSH content of spinal cord mitochondria isolated from hSOD1^G93A^ mutant mice that had been treated with Immunocal^®^ in comparison to mutant mice receiving no supplement, as well as NonTG controls. Mutant mice that did not receive Immunocal^®^ showed marked depletion of mitochondrial GSH levels in comparison to NonTG controls (Figure 5C). Notably, supplementation with Immunocal^®^ did not rescue this effect, indicating that Immunocal^®^ was unable to preserve mitochondrial GSH levels in the spinal cord of hSOD1^G93A^ mice (Figure 5C).

### 3.4. Combination Treatment with Immunocal^®^ and Riluzole Shows Complementary Effects on Disease Onset and Survival in hSOD1^G93A^ Mice

The only current FDA approved treatment for ALS is the anti-glutamatergic drug, riluzole. This compound modestly increases the lifespan of hSOD1^G93A^ mice by approximately 7–10 days, which translates to an increase in the life expectancy of ~2–3 months in human patients [29]. However, despite its effect on lifespan, riluzole has no discernible effect on disease onset in the hSOD1^G93A^ mouse model [30]. Because Immunocal^®^ delayed disease onset, but had no effect on survival, we treated hSOD1^G93A^ mice with a combination of Immunocal^®^ and riluzole to determine if these agents had either complementary or synergistic effects on disease onset and survival when given together.

**Figure 6:**
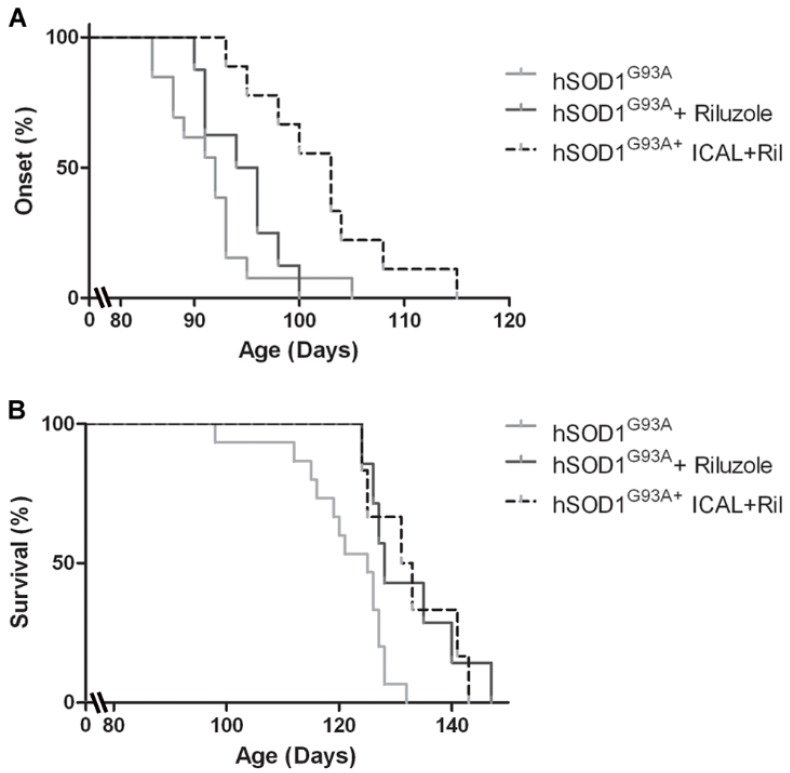
Treatment with Immunocal^®^ an riluzole significantly delays disease onset and extends survival in hSOD1^G93A^ mice. (**A**) hSOD1^G93A^ mice receiving riluzole beginning at 60 days of age (pre-symptomatically) show no delay in disease onset and clinical decline compared to untreated mutant mice (*n* = 8). hSOD1^G93A^ mice receiving riluzole in addition to Immunocal^®^ showed a significant delay in disease onset and clinical decline compared to untreated mutant mice (*p* < 0.001; *n* = 10). Onset curves were compared pair-wise using the Gehan–Breslow–Wilcoxon test. (**B**) hSOD1^G93A^ mice receiving riluzole beginning at 60 days of age (pre-symptomatically) showed a significant extension in survival compared to untreated mutant mice (*n* = 7; *p* < 0.05). Similarly, hSOD1^G93A^ mice receiving riluzole in addition to Immunocal^®^ showed a significant extension in survival compared to untreated mutant mice (*p* < 0.05; *n* = 8). Survival curves were compared pair-wise using the Gehan–Breslow–Wilcoxon test.

Mutant mice receiving riluzole alone did not display a significant difference in disease onset compared to untreated controls (average disease onset occurred at 94.5 ± 1.3 and 91.2 ± 1.2 days of age, respectively); however, mice receiving both Immunocal^®^ and riluzole in combination demonstrated a delay in disease onset of approximately 10 days (average disease onset occurred at 102.1 ± 2.2 days of age; Figure 6A). This delay in disease onset is comparable to that observed in mice treated with Immunocal^®^ alone. Conversely, hSOD1^G93A^ mice treated with riluzole alone showed a modest, but significant increase in survival of approximately seven days with the end-stage occurring at 131.6 ± 2.6 days of age in comparison to untreated mice, which reached the end-stage at 124.9 ± 1.4 days of age (Figure 6B). This modest increase in lifespan is consistent with previous reports using riluzole in the hSOD1^G93A^ mouse model [30]. Mice receiving both riluzole and Immunocal^®^ displayed a similar extension of lifespan, reaching the end-stage at 130.0 ± 3.0 days of age (Figure 6B).

**Figure 7:**
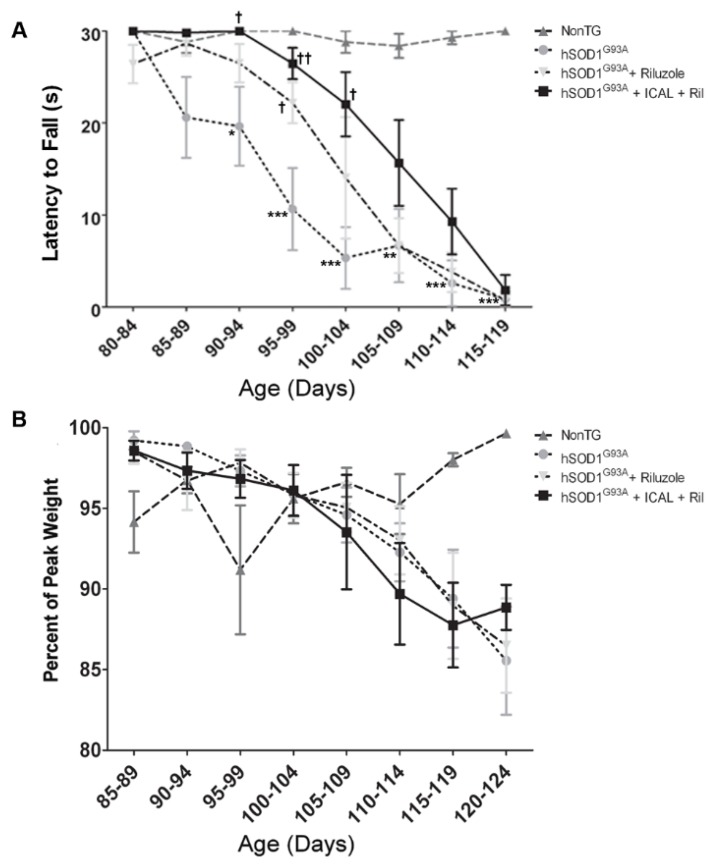
Treatment with Immunocal^®^ and riluzole diminishes the decline in grip strength in hSOD1^G93A^ mice. (**A**) The PaGE hanging wire test expressed as latency to fall at indicated ages (*n* = 8); (**B**) body weight of wild-type and hSOD1^G93A^ mice expressed as percent of peak body weight. All data are represented as the mean ± SEM. *** indicates *p* < 0.001 compared to NonTG; ** indicates *p* < 0.01 compared to NonTG; * indicates *p* < 0.05 compared to NonTG; **^††^** indicates *p* < 0.01 compared to untreated hSOD1^G93A^ mice; and **^†^** indicates *p* < 0.05 compared to untreated hSOD1^G93A^ mice (one-way ANOVA with a post hoc Tukey’s test conducted for each time point).

Moreover, PaGE testing indicated that hSOD1^G93A^ mice that received riluzole in their water displayed a minimal enhancement in grip strength as compared to their untreated counterparts (Figure 7A). However, this effect was accentuated in mice that were supplemented with both Immunocal^®^ and riluzole (Figure 7A). Despite these improvements, however, neither mice receiving riluzole alone nor mice treated with the combination of Immunocal^®^ and riluzole showed any significant change in weight loss compared to untreated mutant controls (Figure 7B). Collectively, these data indicate that mice treated with a combination of riluzole and Immunocal^®^ receive modest benefits of both agents, displaying both a delay in disease onset and increased survival, with preserved grip strength and no overt loss of effect from either agent due to combined administration. While the effects of Immunocal^®^ and riluzole appear to be complementary in this sense, no synergism was observed with combined administration.

## 4. Discussion

GSH depletion occurs in many neurodegenerative diseases, including Alzheimer’s disease and Parkinson’s disease, as well as ALS [13,31,32]. GSH depletion and a decline in antioxidant enzyme activity have been observed in the erythrocytes of sporadic ALS patients with active disease, and interestingly, characteristic pathological changes observed in sporadic disease may be induced by intracellular GSH depletion [12]. For instance, TAR DNA-binding protein-43 (TDP-43) forms cytoplasmic inclusions, which are a hallmark pathology observed in sporadic ALS patients. These inclusions can be recapitulated in cultured neurons subjected to GSH depletion [33]. Moreover, crossing of hSOD1^G93A^ mice with mice lacking the γ-glutamylcysteine ligase regulatory subunit resulted in a significant acceleration of motor neuron disease, a result consistent with GSH depletion being a significant modifier of ALS susceptibility [34]. These cumulative findings suggest a prominent role for GSH depletion in ALS pathogenesis, prompting interest in therapeutically targeting this aspect of the disease.

Here, we investigated the therapeutic potential of Immunocal^®^, a cystine-rich whey protein supplement, in a mouse model of familial ALS. This supplement has been investigated previously as a means to bolster GSH in diseases for which oxidative stress is a significant underlying factor. For instance, oxidative stress is a known activator of human immunodeficiency virus replication, and based on this, Immunocal^®^ was tested as an approach to attenuate this stress and combat infection by increasing intracellular GSH levels [21]. Oxidative stress also plays a significant role in muscle weakness. Oral Immunocal^®^ administration to normal healthy subjects for three months significantly increased muscle power and work capacity in comparison to individuals that received placebo treatment [35]. Immunocal^®^ supplementation also significantly increased lymphocyte GSH levels in these healthy subjects compared to placebo (36% ± 11% increase). Finally, oxidative stress is also believed to play a significant role in the pathogenesis of cystic fibrosis. In cystic fibrosis patients receiving oral Immunocal^®^ for three months, there was an approximately 47% increase in lymphocyte GSH levels compared to individuals receiving a placebo [22]. These studies demonstrate that Immunocal^®^ is capable of bolstering GSH in vivo, although its potential to do so in the central nervous system has not previously been addressed.

Building upon these earlier studies, we have recently found that Immunocal^®^ displays robust neuroprotective activity in vitro against a variety of insults that contribute significantly to oxidative damage within the cell and which are directly relevant to the pathogenesis of multiple neurodegenerative diseases [36]. Moreover, the neuroprotective effects of Immunocal^®^ *in vitro* are blocked by inhibiting GSH synthesis, demonstrating the dependence on enhanced GSH. Consistent with these in vitro findings and those previous observations in various human diseases, we demonstrate here that oral administration of Immunocal^®^ preserved whole blood and spinal cord tissue GSH levels in end-stage hSOD1^G93A^ mice. Transgenic hSOD1^G93A^ mice that did not receive Immunocal^®^ supplementation displayed a marked decrease in the amount of reduced GSH in both whole blood and lumbar spinal cord tissue, when compared to age-matched NonTG control mice. However, transgenic mice administered Immunocal^®^ displayed GSH levels, which were essentially equal to NonTG controls. This preservation of whole blood and spinal cord tissue GSH correlated with a significant delay in the clinical onset of disease and preservation of paw grip strength in this mouse model of familial ALS. Importantly, the ability of Immunocal^®^ to preserve GSH levels in spinal cord indicates that this supplement is capable of acting on the central nervous system without the need for invasive delivery systems, a feature that is highly desirable for potential therapeutic agents in ALS. Since decreases in GSH have been correlated with the rate of disease progression, we conclude that preservation of blood and spinal cord tissue GSH levels with Immunocal^®^ is likely responsible for the observed delay in disease onset [37]. Furthermore, Immunocal^®^ has been shown specifically to attenuate muscle weakness and increase skeletal muscle work power, which may partially account for its ability to maintain paw grip strength in hSOD1^G93A^ mice [35].

Somewhat surprisingly, despite these positive, albeit modest, findings, Immunocal^®^ did not significantly extend survival in hSOD1^G93A^ mice. This result is particularly unexpected given the recent study by Johnson and colleagues, which demonstrated that crossing of hSOD1^G93A^ mice with mice lacking the γ-glutamylcysteine ligase modifier subunit, involved in the rate limiting step of GSH synthesis, significantly accelerated disease progression [34]. Taking these two results together, it seems reasonable to conclude that reducing GSH synthesis is capable of enhancing neuromuscular disease progression; however, preserving whole blood and spinal cord tissue GSH is not sufficient to significantly extend the survival of ALS mice. There is a possibility that the unequal distribution of males to females in the treatment group receiving ad libitum Immunocal^®^ supplementation may have caused the values for survival to be artificially low; however, this is unlikely, as analysis of Immunocal^®^ treated animals in comparison to untreated mutant controls, when separated by gender, revealed that neither males nor females showed a significant extension in survival (end-stage occurred at 122.3 ± 3.0 and 131.0 ± 3.3 days of age for males and females treated with Immunocal^®^, respectively; see Table 2 for values of untreated hSOD1^G93A^ controls). The lack of effect on survival could also be attributed to the multifaceted nature of ALS disease pathology. However, here, we present evidence that the lack of effect on survival may be due more specifically to the inability of Immunocal^®^ to rescue mitochondrial GSH levels in the lumbar spinal cord of ALS mice.

A number of in vitro studies suggest that mutant SOD1 significantly influences mitochondrial GSH and, as a result, susceptibility of motor neuronal cells to MOS. For instance, an *in vitro* study using NSC34 motor neuron-like cells stably transfected with hSOD1^G93A^ demonstrated that cells expressing this mutant form of SOD1 showed markedly and selectively decreased levels of mitochondrial GSH in comparison to parental cells [38]. Similarly, infection of NSC34 cells with adenoviral hSOD1^G93A^ induces oxidative stress, mitochondrial dysfunction and intrinsic apoptosis, which are significantly alleviated by co-expression of the mitochondrial antioxidant enzymes, SOD2 and GSH peroxidase-4 [39]. Finally, NSC34 cells expressing hSOD1^G93A^, but not wild-type (WT) SOD1, show marked morphological and functional alterations in mitochondria and exhibit a significant decrease in the reduced GSH-to-oxidized GSSG ratio in mitochondria following exposure to the inflammatory cytokines, tumor necrosis factor α and interferon γ [40]. Thus, hSOD1^G93A^ appears to sensitize motor neuronal cells *in vitro* to MOS and apoptosis by specifically diminishing the mitochondrial GSH pool.

Moreover, mitochondria lack the enzymes necessary to synthesize and thereby maintain the mitochondrial pool of GSH and rely instead on transporters to import GSH from the cytosol [10,11]. Maintenance of mitochondrial GSH levels by these transporters is critical for cellular survival, as selective depletion of this antioxidant pool by inhibiting GSH transport sensitizes primary neuronal cells to oxidative challenge and subsequent apoptosis [28]. We have previously shown that Bcl-2 is a key regulator of the mitochondrial GSH transport process, and inhibition of Bcl-2 in vitro induces significant GSH depletion at the mitochondria [41]. As Bcl-2 expression is reduced in ALS, mitochondria may experience deficits in GSH transport during disease progression, which may, in turn, exacerbate mitochondrial GSH depletion by hSOD1^G93A^ [42]. Furthermore, mutant SOD1 has been shown to accumulate at mitochondria and form aggregates with Bcl-2, which may further interfere with the ability of Bcl-2 to regulate GSH transport into mitochondria [43]. Consistent with this hypothesis, we demonstrate that mitochondria isolated from the lumbar spinal cord of end-stage hSOD1^G93A^ mutant mice show both a marked decrease in mitochondrial GSH levels and deficits in GSH uptake *in vitro* in comparison to spinal cord mitochondria isolated from NonTG controls. However, while there is evidence to suggest that the ratio of GSH-to-GSSG is reduced at disease onset due to enhanced oxidative stress conditions, which deplete the reduced form of GSH, no statistically significant depletion of total mitochondrial GSH and GSSG was observed at disease onset, suggesting that deficits in transport may not yet be evident at early stages of the disease [14]. Most significantly, hSOD1^G93A^ mice treated with Immunocal^®^ showed no rescue of lumbar spinal cord mitochondrial GSH levels when compared to untreated transgenic mice at end-stage. Thus, while Immunocal^®^ supplementation is sufficient to preserve tissue GSH levels in the spinal cord of ALS mice, this GSH appears unable to enter the mitochondria, where it would normally act to mitigate organelle damage caused by aberrant ROS generation. We propose this scenario as a plausible reason for the lack of extension in the survival of mutant hSOD1^G93A^ mice administered Immunocal^®^ for two reasons.

Firstly, decreased the levels of Bcl-2, and inhibitory interactions between Bcl-2 and mutant SOD1 may explain some deficits in GSH transport into mitochondria due to impaired regulation of this process. However, there are also other possibilities to consider, which have not been previously explored in the context of ALS. For example, decreases in GSH transport from the cytosol to the mitochondria could be compromised at the level of the mitochondrial GSH transporters themselves. This may occur if proteins, such as the dicarboxylate carrier or the 2-oxyglutarate carrier, both of which play a major role in maintaining mitochondrial GSH levels in brain tissue, are damaged or modified by oxidative species generated within the mitochondria [11,28,41]. Indeed, disruption of the overall architecture of key GSH transport machinery under conditions of mitochondrial oxidative stress could interfere with or abrogate their ability to move GSH from the cytosol to the mitochondria by abolishing the transport function of these proteins or preventing their interaction with key regulators, such as Bcl-2. The 2-oxyglutarate carrier, for example, possesses three critical cysteine residues that are vital for its transport function and could be sensitive to oxidative modification [44]. Therefore, even in conditions where GSH production is enhanced and cytosolic levels of GSH are elevated, such as with Immunocal^®^, the rescue of the mitochondrial GSH pool may not be possible without first correcting impairments in the mitochondrial glutathione transport machinery.

Secondly, deficits in glutathione transport into the mitochondria could contribute to the adoption of an inflammatory phenotype by surrounding glial cells. Neuroinflammation is a contributing factor to many neurodegenerative disorders and is known to play a significant role in the pathology and progression of ALS [45,46]. Interestingly, GSH depletion results in activation of both astrocytes and microglia due to increased levels of oxidative stress within these cell types and results in secretion of a number of pro-inflammatory and neurotoxic factors [47,48]. The reverse is also true: transition to an inflammatory phenotype through stimulation by agents, such as lipopolysaccharide, also results in GSH depletion and induction of MOS [49]. Moreover, GSH depletion in glial cells causes upregulation of the x_C_^−^ Sx_C_^−^ transporter, which is responsible for antiporting glutamate for cystine to stimulate GSH production [50,51]. Increased secretion of glutamate can then contribute to excitotoxicity and neuronal death. Inflammation in both astrocytes and microglia can be mitigated by supplementation with GSH, GSH precursors or protection of the critical mitochondrial GSH pool, which reduce oxidative stress within these cells and promote the maintenance of a neuroprotective phenotype [48,51,52]. Although no data exists suggesting that Immunocal^®^ specifically is effective at preventing neuroinflammation, these studies do suggest that increasing GSH levels in glial cells through precursor supplementation may be a viable therapeutic strategy. However, the positive effects of GSH supplementation appear to be at least partially dependent on the ability of GSH to prevent oxidative damage at the level of the mitochondria [49,52]. Thus, mitochondrial GSH transport deficits in ALS, such as those observed in this study, may render supplements, such as Immunocal^®^, ineffective at attenuating glial inflammation and subsequent neuronal cell death.

Because Immunocal^®^ delayed disease onset, but had no effect on survival, this whey supplement was tested in combination with the anti-glutamatergic compound, riluzole, which displays the opposite effect [30]. As expected, mice treated with riluzole alone displayed a significant extension in survival in comparison to untreated mutant mice, but no effect on disease onset. Mice treated in combination with both agents displayed modest benefits of both Immunocal^®^ and riluzole, developing neuromuscular deficits at a later time point and showing a modest increase in overall survival. Additionally, these results demonstrate that there are no overt adverse effects from the combined administration of these agents, an important consideration when combining therapeutic treatments. These data also show that Immunocal^®^ administered at a known dosage of approximately 660 mg/kg/day recapitulates the modest beneficial effects observed in mutant mice that were given ad libitum access to Immunocal^®^ in their drinking water. For the average adult, this dosage would be equivalent to consuming about four to five packets of Immunocal^®^ each day, or about twice the manufacturer’s recommended daily value.

The effect of Immunocal^®^ on delaying disease onset in this ALS mouse model is quite modest. In pre-clinical studies, such as these, it has been recommended that a relatively large number of animals should be tested (~25 per group) to have confidence in the observation of such a small effect [53]. In Table 3, we provide a breakdown of each of the Immunocal^®^ dosing regimens used in this study. In every case, whether Immunocal^®^ was administered ad libitum in drinking water (Group 3) or by oral gavage, either alone (Group 4) or in combination with riluzole (Group 5), we observed a statistically significant delay in disease onset of 7–10 days. Moreover, the mean onset was not significantly different amongst any of the Immunocal^®^ treatment groups, regardless of the method of dosing or whether or not the supplement was given in combination with riluzole (which is known to have no effect on disease onset in the ALS mouse model) [30]. Thus, the data taken as a whole demonstrate a reproducible and statistically significant delay in disease onset with Immunocal^®^ treatment.

Finally, vitamin E displays essentially the same effect in this mouse model of ALS, producing a modest delay in disease onset, but no significant extension of survival [30]. Nonetheless, despite its very modest effects in the ALS mouse model, a pooled analysis of five prospective cohort studies suggests that long-term supplementation with vitamin E is associated with lower ALS rates [54,55]. Thus, even a modest effect in the ALS mouse model may translate into a measurable beneficial effect for some subpopulation of patients, particularly when one considers long-term use with nutritional supplements, such as vitamin E, creatine or Immunocal^®^. 

## 5. Conclusions

Despite its lack of effect on overall survival, we found that Immunocal^®^ administered orally to hSOD1^G93A^ mice significantly reduced age-associated decline in skeletal muscle function, as measured by the PaGE test. Moreover, Immunocal^®^ significantly delayed the clinical onset of neuromuscular disease in these mutant mice. Finally, Immunocal^®^ prevented depletion of whole blood and spinal cord tissue GSH and maintained the GSH/GSSG ratio at levels indistinguishable from age-matched NonTG mice. Thus, Immunocal^®^ supplementation had a significant, albeit modest, beneficial effect in the hSOD1^G93A^ mutant mouse model of ALS that was positively correlated with the preservation of whole blood and spinal cord tissue GSH levels. Unfortunately, Immunocal^®^ failed to rescue mitochondrial GSH levels in spinal cord of end-stage hSOD1^G93A^ mice. This latter result provides a plausible molecular mechanism for the observed lack of effect of this supplement on overall survival in ALS mice. Moreover, these findings suggest significant limitations to the potential utility of this supplement as a therapeutic agent for ALS. Future studies aimed at elucidating the mechanisms related to dysfunctional GSH transport in spinal cord mitochondria of ALS mice are necessary to further evaluate the pre-clinical effects of Immunocal^®^ in this disease.
